# Joint association of hyperuricemia and chronic kidney disease with mortality in patients with chronic heart failure

**DOI:** 10.3389/fendo.2023.1131566

**Published:** 2023-04-05

**Authors:** Chi Wang, Hebin Che, You Zhou, Ruiqing Wang, Di Zhu, Liting Cheng, Chongyou Rao, Qin Zhong, Zongren Li, Yongjie Duan, Jiayu Xu, Wei Dong, Yongyi Bai, Kunlun He

**Affiliations:** ^1^ Graduate School of People's Liberation Army General Hospital, Beijing, China; ^2^ Medical Big Data Research Center, Medical Innovation Research Department of People's Liberation Army General Hospital, Beijing, China; ^3^ School of Medicine, Nankai University, Tianjin, China; ^4^ Department of Cardiology, Sixth Medical Center of People's Liberation Army General Hospital, Beijing, China; ^5^ Department of Cardiology, Second Medical Center of People's Liberation Army General Hospital, Beijing, China

**Keywords:** uric acid, chronic kidney disease, heart failure, mortality risk, cohort study

## Abstract

**Background:**

The joint association of hyperuricemia and chronic kidney disease (CKD) with mortality in patients with chronic heart failure (CHF) is not conclusive.

**Methods:**

This retrospective cohort study was conducted in Chinese People's Liberation Army General Hospital, Beijing, China. We included 9,367 patients with CHF, who were hospitalized between January 2011 and June 2019. The definitions of hyperuricemia and CKD were based on laboratory test, medication use, and medical record. We categorized patients with CHF into 4 groups according to the absence (-) or presence (+) of hyperuricemia and CKD. The primary outcomes included in-hospital mortality and long-term mortality. We used multivariate logistic regression and Cox proportional hazards regression to estimate the mortality risk according to the hyperuricemia/CKD groups.

**Results:**

We identified 275 cases of in-hospital mortality and 2,883 cases of long-term mortality in a mean follow-up of 4.81 years. After adjusting for potential confounders, we found that compared with the hyperuricemia-/CKD- group, the risks of in-hospital mortality were higher in the hyperuricemia+/CKD- group (odds ratio [OR], 95% confidence interval [CI]: 1.58 [1.01-2.46]), hyperuricemia-/CKD+ group (OR, 95% CI: 1.67 [1.10-2.55]), and hyperuricemia+/CKD+ group (OR, 95% CI: 2.12 [1.46-3.08]). Similar results were also found in long-term mortality analysis. Compared with the hyperuricemia-/CKD- group, the adjusted hazard ratios and 95% CI for long-term mortality were 1.25 (1.11-1.41) for hyperuricemia+/CKD- group, 1.37 (1.22-1.53) for hyperuricemia-/CKD+ group, and 1.59 (1.43-1.76) for hyperuricemia+/CKD+ group. The results remained robust in the sensitivity analysis.

**Conclusions:**

Hyperuricemia and CKD, both individually and cumulatively, are associated with increased mortality risk in patients with CHF. These results highlighted the importance of the combined control of hyperuricemia and CKD in the management of heart failure.

## Introduction

Chronic heart failure (CHF) is one of the major causes of mortality and affects 1%-2% adults worldwide (1, 2). Approximately 74% of CHF patients have at least one non-cardiac comorbidity (3), which increase the medical complexity and mortality rates. Chronic kidney disease (CKD) is a progressive condition characterized by decreased estimated glomerular filtration rate (eGFR) and/or presence of proteinuria. More than 50% of deaths in patients with CKD are resulted from cardiovascular disease (4). CKD is highly prevalent in CHF with the comorbidity rate ranging from 33% to 48% (3, 5, 6). A meta-analysis including 57 studies concluded that patients with the cooccurrence of CKD and CHF experienced nearly 2-fold higher risk of mortality (7). Hyperuricemia is one of the major metabolic diseases that caused by both an increased production and a decreased excretion of uric acid (UA). Various studies have shown that hyperuricemia was associated with higher risk of mortality in general population and patients with cardiovascular disease (8–10). Hyperuricemia affects about half of patients with CHF (11), and accounts for increased risk of hospitalization, cardiovascular mortality, and all-cause mortality regardless of the ejection fraction (EF) phenotype of CHF (12, 13).

Hyperuricemia is a common finding in CKD due to reduced excretion of UA (14), and also an independent risk factor for the development of CKD (15). The comorbidity of hyperuricemia and CKD poses worse outcomes in general population (16–18). Although, similar findings were not generated in several studies based on patients with CHF (19, 20). In the Beta-Blocker Evaluation of Survival Trial, hyperuricemia is associated with hospitalization and mortality in CHF patients without CKD, but not those with CKD (19). In addition, CHART-2 study and EVEREST study demonstrated that elevated serum uric acid is not associated with mortality in CHF patients with eGFR <60ml/min/1.73m^2^ and CHF patients with eGFR <30ml/min/1.73m^2^ (20, 21). However, these previous studies did not account for proteinuria in the definition of CKD, which have limited information on the association between hyperuricemia, CKD, and adverse outcomes. Moreover, their findings should be validated in other populations because the characteristics of heart failure vary greatly across different regions and ethnicities (22, 23). Therefore, the aim of our study was to comprehensively examine the joint association of hyperuricemia and CKD with in-hospital mortality and long-term mortality in a population of hospitalized CHF patients in China.

## Materials and methods

### Study design and patients

This retrospective cohort study was conducted in the Chinese People’s Liberation Army General Hospital, a large-scale tertiary hospital in Beijing, China. We used the *International Classification of Diseases* (I110, I130, I132, I42, I43, I50), *Tenth Revision*, for the identification of potential heart failure patients. A panel of 3 physicians reviewed patient medical records to confirm the diagnosis of CHF. CHF was diagnosed according to the criteria of *2021 European Society of Cardiology Guidelines for the diagnosis and treatment of acute and chronic heart failure* (1), and further subclassified into heart failure with reduced ejection fraction (HFrEF), heart failure with mildly reduced ejection fraction (HFmrEF), and heart failure with preserved ejection fraction (HFpEF) according to the EF value reported on echocardiography. The study protocol complied with the Declaration of Helsinki and was approved by the ethics committee of the Chinese People’s Liberation Army General Hospital (Number: S2018-269-02).

### Data collection and definitions

Data on demographic characteristics, anthropometric measurements, laboratory tests, medication use during hospitalization, and medication use after discharge were abstracted from the hospital electronic medical record database. Demographic characteristics included age and sex. Anthropometric measurements included height, weight, systolic blood pressure (SBP), diastolic blood pressure (DBP), and heart rate. Body mass index was calculated as weight (kg) divided by the square of height (m). Laboratory tests included measurements of hemoglobin, blood glucose, serum creatinine, UA, N-terminal pro-B-type natriuretic peptide (NT-proBNP), C-reactive protein (CRP), glycated hemoglobin (HbA1c), urinary albumin-to-creatinine ratio (ACR), and dipstick proteinuria. eGFR was calculated using the *Chronic Kidney Disease Epidemiology Collaboration creatinine equation* (24). For patients who underwent repeated laboratory tests, we only included the result of the first test in our analyses. Anti-CHF medications included renin-angiotensin-aldosterone system inhibitors (RAASi), spironolactone, diuretics (including loop diuretics, thiazide diuretics, and tolvaptan), beta-blockers, digitalis, and nitrates.

Hyperuricemia was defined as serum UA >420 μmol/L, use of UA-lowering medication, or physician-diagnosed hyperuricemia (25–27). CKD was defined as eGFR <60 mL/min/1.73m^2^, ACR ≥30 mg/g, proteinuria ≥2+ on dipstick, or physician-diagnosed CKD (28, 29). Other comorbidities, including myocardial infarction, hypertension, atrial fibrillation, valvular heart disease, thrombotic complication, diabetes, stroke, anemia, chronic pulmonary disease, liver disease, connective tissue disease, and cancer were identified based on the medical record. Diabetes was further identified by fasting blood glucose ≥7 mmol/L, random blood glucose ≥11.1 mmol/L, HbA1c ≥6.5%, or use of hypoglycemic medication (30). Anemia was further identified by hemoglobin <130 g/L in men, hemoglobin <120 g/L in women, or use of iron or erythropoietin therapy (6).

### Assessment of outcomes

The outcome of the current study were in-hospital mortality and long-term mortality. Information on in-hospital mortality was checked by reviewing medical records. For those patients without in-hospital mortality, information on long-term mortality was obtained by checking subsequent medical records and telephonic interview. The cause of mortality (cardiovascular [CV] mortality or not) was asked during the telephonic interview.

### Statistical analysis

We divided the study patients into 4 groups according to the absence (-) or presence (+) of hyperuricemia and CKD: hyperuricemia-/CKD-, hyperuricemia+/CKD-, hyperuricemia-/CKD+, and hyperuricemia+/CKD+. Continuous variables with a normal distribution are presented as mean ± standard deviation; those with a non-normal distribution are presented as median (interquartile range). Continuous variables were compared using one-way analysis of variance or the Kruskal-Wallis test as appropriate. Categorical variables are presented as numbers with percentage and were compared using the chi square test. Risk of in-hospital mortality was estimated using multivariate logistic regression to calculate odds ratios (ORs) with 95% confidence intervals (CIs). Cox proportional hazards regression was used to investigate the association between exposures (hyperuricemia/CKD groups) and long-term mortality by calculating hazard ratios (HRs) with 95% CIs. The proportional hazard assumption was checked using Schoenfeld residuals. The fully adjusted model included age, sex, BMI, SBP, DBP, heart rate, EF phenotype, NT-proBNP concentration, CRP concentration, myocardial infarction, hypertension, atrial fibrillation, valvular heart disease, thrombotic complication, diabetes, stroke, anemia, chronic pulmonary disease, liver disease, connective tissue disease, cancer, and medication use of RAASi, spironolactone, diuretics, beta-blockers, digitalis, and nitrates. Missing values were handled by multiple imputation.

A recent study suggested that hyperuricemia with crystalluria, but not asymptomatic hyperuricemia, was associated with progression of CKD (31). Therefore, we performed analyses examining the combined effect of CKD with asymptomatic hyperuricemia and gout, respectively. Given that the pathophysiology of reduced eGFR is different from that of proteinuria, we performed analyses defining CKD as only eGFR <60ml/min/1.73m^2^ and only proteinuria, respectively. To test the robustness and consistency of our findings, we performed a sensitivity analysis using serum UA >420 μmol/L in men and >360 μmol/L in women as the cutoff points for hyperuricemia (32). To reduce the possibility of reverse causation in the long-term mortality analysis, we excluded patients who died within the 6 months of follow-up in a sensitivity analysis. To investigate the joint impact of hyperuricemia and CKD on CV mortality, we performed analyses using CV mortality as the outcome event.

To better understand the interaction between serum UA and renal dysfunction, we further performed subanalyses with patients grouped by serum UA level (≤420 μmol/L, 421-600 μmol/L, >600 μmol/L) (25, 27, 33, 34) and stratified by CKD and CKD subgroups (eGFR ≥90, 60-89, 30-59, <30ml/min/1.73m^2^; with and without proteinuria) (35), and subanalyses with patients grouped by risk classification of CKD progression (low, moderate, high, and very high risk) (35) and stratified by hyperuricemia. To explore whether the association between hyperuricemia/CKD groups and mortality in CHF patients could be modified by age (≥65 and <65 years), sex, myocardial infarction, hypertension, EF phenotype (HFrEF, HFmrEF and HFpEF), and New York Heart Association (NYHA) classification (I-II, III, and IV), subgroup analyses by these factors were performed.

Statistical analyses were conducted using SAS version 9.4 (SAS Institute, Cary, NC, USA). Two-sided *P <*0.05 was considered significant.

## Results

**Figure 1:**
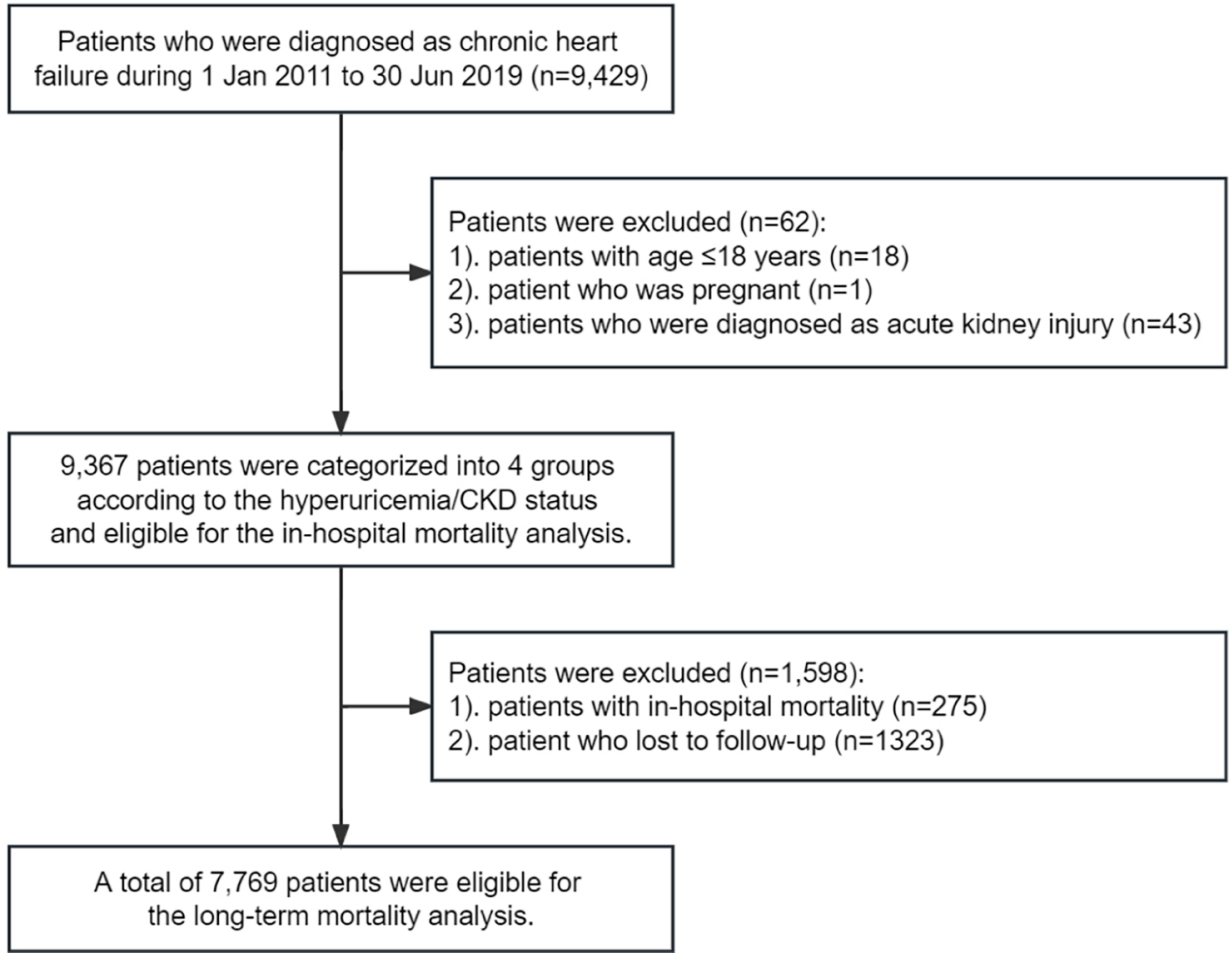
Eligibility of the study patients. The flowchart of the eligibility of 9,367 patients included in the in-hospital mortality analysis, and 7,769 patients included in the long-term mortality analysis.

In the current study, we identified 9,429 CHF patients who were hospitalized between 1 January 2011 and 30 June 2019. 18 patients were excluded due to the age of <18 years, 1 patient was excluded due to pregnancy, and 43 patients were excluded due to the diagnosis of acute kidney injury. A total of 9,367 CHF patients were included in the in-hospital mortality analysis (**Figure 1**). Of these, the mean age was 62.64 ± 14.61 years, 6,244 (66.66%) were men, 275 (2.94%) had in-hospital mortality (182 were CV mortality), and 1,323 (14.12%) lost to follow-up. Finally, a total of 7,769 CHF patients were included in the long-term mortality analysis (**Figure 1**). The patients lost to follow-up were younger, and had a lower proportion of hyperuricemia but similar proportion of CKD (**Supplementary Table 1**). Patients in the hyperuricemia+/CKD- group were younger and more likely to be men. They also had higher BMI, heart rate, and higher prevalence of HFrEF, atrial fibrillation, and valvular heart disease, while patients in the hyperuricemia-/CKD+ group had higher blood pressure, and higher prevalence of diabetes, stroke, anemia, chronic pulmonary disease, and connective tissue disease. Those in the hyperuricemia+/CKD+ group had higher serum concentration of NT-proBNP and higher prevalence of NYHA class IV heart failure (**Table 1**). Patients included in the long-term mortality analysis exhibited similar characteristics (**Supplementary Table 2**).

**Table 1:** Characteristics of 9,367 patients with CHF according to hyperuricemia/CKD groups.

Variables	Hyperuricemia- /CKD-	Hyperuricemia+ /CKD-	Hyperuricemia- /CKD+	Hyperuricemia+ /CKD+	*P* value
No. of patients	4,198	1,712	1,355	2,102	
Age, mean ± SD, y	62.82 ± 13.15	55.92 ± 13.79	67.67 ± 15.10	64.50 ± 15.75	<0.01
Men, No. (%)	2,621 (62.43)	1,383 (80.78)	783 (57.79)	1,457 (69.31)	<0.01
BMI, mean ± SD, kg/m^2^	24.80 ± 3.86	25.76 ± 4.56	24.55 ± 4.07	25.18 ± 4.47	<0.01
SBP, mean ± SD, mmHg	128.12 ± 20.07	123.38 ± 20.39	139.95 ± 25.10	134.35 ± 24.77	<0.01
DBP, mean ± SD, mmHg	74.40 ± 12.56	75.97 ± 14.12	77.14 ± 14.76	76.77 ± 16.13	<0.01
Heart Rate, mean ± SD, bpm	81.74 ± 17.62	84.68 ± 18.46	83.68 ± 18.22	84.05 ± 19.32	<0.01
EF phenotypes, No. (%)					<0.01
HFrEF	1,515 (36.08)	904 (52.80)	388 (28.63)	828 (39.39)	
HFmrEF	964 (22.96)	292 (17.06)	301 (22.21)	404 (19.22)	
HFpEF	1,719 (40.94)	516 (30.14)	666 (49.15)	870 (41.39)	
NYHA class, No. (%)					<0.01
I-II	2,139 (50.95)	610 (35.63)	436 (32.18)	519 (24.69)	
III	1,651 (39.32)	824 (48.13)	679 (50.11)	1,057 (50.29)	
IV	408 (9.72)	278 (16.24)	240 (17.71)	526 (25.02)	
Laboratory test					
UA, mean ± SD, μmol/L	313.26 ± 65.59	511.72 ± 92.53	329.24 ± 66.94	552.72 ± 126.67	<0.01
eGFR, median (IQR), ml/min/1.73m^2^	87.71 (76.54, 97.22)	82.99 (71.75, 95.26)	49.01 (23.83, 59.71)	39.23 (20.04, 53.65)	<0.01
NT-proBNP, median (IQR), pg/mL	1224.0 (553.0, 2776.0)	1905.0 (805.0, 4350.0)	4570.0 (1719.0, 14702.0)	6106.5 (2318.0, 17351.0)	<0.01
CRP, No. (%)					<0.01
<10 mg/L	2,752 (78.05)	1,155 (78.09)	833 (66.43)	1,262 (65.59)	
10-30 mg/L	380 (10.77)	205 (13.86)	219 (17.46)	376 (19.54)	
≥30 mg/L	394 (11.17)	119 (8.05)	202 (16.11)	286 (14.86)	
Proteinuria, No. (%)	0 (0)	0 (0)	579 (46.47)	786 (39.92)	<0.01
Comorbidities					
Myocardial infarction, No. (%)	1,560 (37.16)	478 (27.92)	493 (36.38)	663 (31.54)	<0.01
Hypertension, No. (%)	2,212 (52.69)	817 (47.72)	1,053 (77.71)	1,586 (75.45)	<0.01
Atrial fibrillation, No. (%)	1,275 (30.37)	574 (33.53)	367 (27.08)	654 (31.11)	<0.01
Valvular heart disease, No. (%)	1,083 (25.80)	446 (26.05)	190 (14.02)	395 (18.79)	<0.01
Thrombotic complications, No. (%)	215 (5.12)	101 (5.90)	89 (6.57)	123 (5.85)	0.19
Gout, No. (%)	0 (0)	68 (3.97)	0 (0)	138 (6.57)	<0.01
Diabetes, No. (%)	1,458 (34.73)	532 (31.07)	743 (54.83)	1,042 (49.57)	<0.01
Stroke, No. (%)	783 (18.65)	230 (13.43)	386 (28.49)	463 (22.03)	<0.01
Anemia, No. (%)	1,152 (27.44)	358 (20.91)	839 (61.92)	1,217 (57.90)	<0.01
Chronic pulmonary diseases, No. (%)	501 (11.93)	184 (10.75)	227 (16.75)	316 (15.03)	<0.01
Liver diseases, No. (%)	529 (12.60)	240 (14.02)	203 (14.98)	340 (16.18)	<0.01
Connective tissue diseases, No. (%)	58 (1.38)	23 (1.34)	37 (2.73)	49 (2.33)	<0.01
Cancer, No. (%)	165 (3.93)	46 (2.69)	51 (3.76)	62 (2.95)	0.05
Medication during hospitalization					
UA-lowering agents, No. (%)	0 (0)	108 (6.31)	0 (0)	311 (14.80)	<0.01
RAASi, No. (%)	2,368 (56.41)	1,070 (62.50)	749 (55.28)	1,141 (54.28)	<0.01
Spironolactone, No. (%)	2,916 (69.46)	1,434 (83.76)	845 (62.36)	1,471 (69.98)	<0.01
Diuretics, No. (%)	3,071 (73.15)	1,464 (85.51)	1,092 (80.59)	1,921 (91.39)	<0.01
Beta-blockers, No. (%)	3,256 (77.56)	1,426 (83.29)	1,072 (79.11)	1,689 (80.35)	<0.01
Digitalis, No. (%)	1,918 (45.69)	1,032 (60.28)	465 (34.32)	961 (45.72)	<0.01
Nitrates, No. (%)	2,553 (60.81)	959 (56.02)	965 (71.22)	1,450 (68.98)	<0.01

BMI, body mass index; bpm, beats per minute; CHF, chronic heart failure; CKD, chronic kidney disease; CRP, C-reactive protein; DBP, diastolic blood pressure; eGFR, estimated glomerular filtration rate; IQR, interquartile range; NT-proBNP, N-terminal pro-B-type natriuretic peptide; RAASi, renin–angiotensin–aldosterone system inhibitors; SBP, systolic blood pressure; SD, standard deviation; UA, uric acid.

Hyperuricemia+/CKD+ group had the highest incidence rate of in-hospital mortality (5.80%), followed by hyperuricemia-/CKD+ group (4.28%), hyperuricemia+/CKD- group (2.10%), and hyperuricemia-/CKD- group (1.41%) (**Table 2**). After adjusting for potential confounders, the risk of in-hospital mortality was twice as high in the hyperuricemia+/CKD+ group compared with the hyperuricemia-/CKD- group (OR [95% CI], 2.12 [1.46-3.08]). Those with hyperuricemia alone and CKD alone also exhibited a significantly higher risk of in-hospital mortality compared with the hyperuricemia-/CKD- group (OR [95% CI], 1.58 [1.01-2.46] and 1.67 [1.10-2.55], respectively) (**Table 2**). Analyses of asymptomatic hyperuricemia and gout, defining CKD as only proteinuria, using serum UA >420 μmol/L in men and >360 μmol/L in women as the cutoff point to define hyperuricemia all generated similar results (**Table 2**). Similar findings were also observed in the CV mortality analyses (**Supplementary Table 3**).

**Table 2:** Risk of in-hospital mortality and long-term mortality according to hyperuricemia/CKD groups.

	Hyperuricemia/CKD Groups			
	Hyperuricemia- /CKD-	Hyperuricemia+ /CKD-	Hyperuricemia- /CKD+	Hyperuricemia+ /CKD+
In-hospital mortality, OR (95% CI)				
Cases, No.	59	36	58	122
Incidence rate, %	1.41	2.10	4.28	5.80
Model 1	1 (Reference)	1.51 (0.99-2.29)	3.14 (2.17-4.53)	4.32 (3.15-5.93)
Model 2	1 (Reference)	1.91 (1.25-2.92)	2.61 (1.80-3.78)	4.00 (2.91-5.50)
Model 3	1 (Reference)	1.58 (1.01-2.46)	1.67 (1.10-2.55)	2.12 (1.46-3.08)
Model 4	1 (Reference)	1.57 (1.00-2.45)	1.65 (1.08-2.51)	2.05 (1.41-3.00)
Model 5	1 (Reference)	1.64 (0.22-12.48)	1.68 (1.05-2.68)	2.82 (1.22-6.53)
Model 6	1 (Reference)	2.12 (1.44-3.11)	1.29 (0.83-2.02)	1.49 (1.03-2.17)
Model 7	1 (Reference)	1.51 (1.08-2.10)	1.77 (1.04-3.03)	2.51 (1.58-3.99)
Model 8	1 (Reference)	1.40 (0.91-2.16)	1.62 (1.04-2.53)	2.05 (1.40-2.98)
Long-term mortality, HR (95% CI)				
Cases, No.	1,020	420	571	872
Incidence rate, per 1000-person-year	54.15	58.50	121.06	131.26
Model 1	1 (Reference)	1.08 (0.96-1.21)	2.20 (1.98-2.44)	2.37 (2.16-2.59)
Model 2	1 (Reference)	1.32 (1.18-1.48)	1.84 (1.66-2.04)	2.23 (2.03-2.44)
Model 3	1 (Reference)	1.25 (1.11-1.41)	1.37 (1.22-1.53)	1.59 (1.43-1.76)
Model 4	1 (Reference)	1.28 (1.14-1.44)	1.37 (1.22-1.53)	1.59 (1.43-1.77)
Model 5	1 (Reference)	0.70 (0.39-1.24)	1.31 (1.16-1.48)	1.44 (1.09-1.90)
Model 6	1 (Reference)	1.26 (1.13-1.41)	1.38 (1.23-1.56)	1.56 (1.41-1.73)
Model 7	1 (Reference)	1.39 (1.27-1.52)	1.53 (1.31-1.78)	1.55 (1.34-1.80)
Model 8	1 (Reference)	1.16 (1.04-1.30)	1.32 (1.17-1.48)	1.57 (1.42-1.74)
Model 9	1 (Reference)	1.28 (1.13-1.45)	1.41 (1.25-1.59)	1.61 (1.44-1.80)

CKD, chronic kidney disease; CI, confidence interval; HR, hazard ratio; OR, odds ratio.

Model 1 was crude model. Model 2 was adjusted for age and sex. Model 3 was further adjusted for BMI, SBP, DBP, heart rate, EF phenotype, NT-proBNP, CRP, myocardial infarction, hypertension, atrial fibrillation, valvular heart disease, thrombotic complication, diabetes, stroke, anemia, chronic pulmonary disease, liver disease, connective tissue disease, cancer, and medication use of RAASi, spironolactone, diuretics, beta-blockers, digitalis, and nitrates. Model 4 was the analysis examining the combined effect of asymptomatic hyperuricemia and CKD. Model 5 was the analysis examining the combined effect of gout and CKD. Model 6 defined CKD as eGFR <60ml/min/1.73m^2^. Model 7 defined CKD as proteinuria. Model 8 was sensitivity analysis using serum uric acid ≥420 μmol/L in men and ≥360 μmol/L in women as another cutoff value for the definition of hyperuricemia. Model 9 was sensitivity analysis excluding those who died within the 6 months of follow-up.

During the mean follow-up of 4.81 years, a total of 2,883 deaths were identified. Among them, 1,433 were CV death, 1,059 were non-CV death, and 391 deaths could not be classified due to lack of information. The hyperuricemia+/CKD+ group had the highest incidence rate of long-term mortality among all 4 groups (**Table 2**). Compared with the hyperuricemia-/CKD- group, the adjusted HRs and 95% CI for long-term mortality were 1.25 (1.11-1.41) for the hyperuricemia+/CKD- group, 1.37 (1.22-1.53) for the hyperuricemia-/CKD+ group, and 1.59 (1.43-1.76) for the hyperuricemia+/CKD+ group (**Table 2**). The results did not materially change in analyses of asymptomatic hyperuricemia and gout, analyses defining CKD as only reduced eGFR and only proteinuria, sensitivity analyses, and CV mortality analyses (**Table 2** and **Supplementary Table 3**).

The long-term mortality risk was proportional to serum UA level, regardless of the CKD status or CKD subtypes (**Table 3**). Although, further analyses showed that the association of serum UA with in-hospital mortality was more evident in patients with eGFR ≥60 ml/min/1.73m^2^ (*P*-interaction =0.02), it was not significant in patients with eGFR <60ml/min/1.73m^2^ (**Table 3**). The association with long-term mortality was more evident in patients without proteinuria compared with those with proteinuria (*P*-interaction =0.02) (**Table 3**). Furthermore, the in-hospital and long-term mortality risk increased with the upgrade of risk classification of CKD progression, and did not vary between patients with and without hyperuricemia (*P*-interaction =0.29 for in-hospital mortality and 0.55 for long-term mortality) (**Table 4**).

**Table 3:** Mortality risk grouped by serum UA level and stratified by CKD and CKD subtypes.

	Serum UA level			*P-*trend	*P*-interaction
	≤420μmol/L	420-600μmol/L	>600μmol/L		
In-hospital mortality, OR (95% CI)					
CHF without CKD	1 (Reference)	1.19 (0.70-2.01)	2.51 (1.19-5.30)	0.04	0.20
CHF with CKD	1 (Reference)	1.22 (0.82-1.80)	1.75 (1.11-2.78)	0.02	
CHF with eGFR ≥90ml/min/1.73m^2^	1 (Reference)	0.79 (0.34-1.86)	4.97 (1.57-15.74)	0.13	0.02
CHF with eGFR 60-89ml/min/1.73m^2^	1 (Reference)	2.19 (1.26-3.84)	4.54 (2.08-9.90)	<0.01	
CHF with eGFR 30-59ml/min/1.73m^2^	1 (Reference)	1.16 (0.63-2.14)	1.67 (0.78-3.61)	0.21	
CHF with eGFR <30ml/min/1.73m^2^	1 (Reference)	1.25 (0.62-2.51)	1.81 (0.84-3.92)	0.14	
CHF without proteinuria	1 (Reference)	1.36 (0.94-1.96)	1.65 (1.02-2.68)	0.03	0.35
CHF with proteinuria	1 (Reference)	1.30 (0.68-2.49)	2.79 (1.26-6.15)	0.02	
Long-term mortality, HR (95% CI)					
CHF without CKD	1 (Reference)	1.15 (1.01-1.31)	1.53 (1.20-1.96)	<0.01	0.29
CHF with CKD	1 (Reference)	1.15 (1.02-1.29)	1.41 (1.21-1.66)	<0.01	
CHF with eGFR ≥90ml/min/1.73m^2^	1 (Reference)	1.04 (0.83-1.30)	1.74 (1.11-2.72)	0.10	0.42
CHF with eGFR 60-89ml/min/1.73m^2^	1 (Reference)	1.25 (1.07-1.45)	1.66 (1.27-2.19)	<0.01	
CHF with eGFR 30-59ml/min/1.73m^2^	1 (Reference)	1.14 (0.97-1.33)	1.37 (1.09-1.73)	<0.01	
CHF with eGFR <30ml/min/1.73m^2^	1 (Reference)	1.17 (0.95-1.44)	1.38 (1.07-1.78)	0.01	
CHF without proteinuria	1 (Reference)	1.29 (1.17-1.43)	1.59 (1.36-1.85)	<0.01	0.02
CHF with proteinuria	1 (Reference)	1.06 (0.87-1.28)	1.40 (1.04-1.89)	0.06	

CHF, chronic heart failure; CI, confidence interval; CKD, chronic kidney disease; eGFR, estimated glomerular filtration rate; HR, hazard ratio; OR, odds ratio; UA, uric acid.

Models were adjusted for age, sex, BMI, SBP, DBP, heart rate, EF phenotype, NT-proBNP, CRP, myocardial infarction, hypertension, atrial fibrillation, valvular heart disease, thrombotic complication, diabetes, stroke, anemia, chronic pulmonary disease, liver disease, connective tissue disease, cancer, and medication use of RAASi, spironolactone, diuretics, beta-blockers, digitalis, and nitrates.

**Table 4:** Mortality risk grouped by risk classification of CKD progression and stratified by hyperuricemia.

	Risk classification of CKD progression				*P-*trend	*P*-interaction
	Low risk	Moderate risk	High risk	Very high risk		
In-hospital mortality, OR (95% CI)						
CHF without hyperuricemia	1 (Reference)	1.48 (0.86-2.53)	2.03 (1.00-4.12)	2.76 (1.35-5.66)	<0.01	0.29
CHF with hyperuricemia	1 (Reference)	1.16 (0.65-2.05)	1.36 (0.72-2.55)	1.06 (0.57-1.98)	0.84	
Long-term mortality, HR (95% CI)						
CHF without hyperuricemia	1 (Reference)	1.22 (1.07-1.39)	1.40 (1.16-1.71)	1.80 (1.48-2.19)	<0.01	0.55
CHF with hyperuricemia	1 (Reference)	1.28 (1.08-1.52)	1.38 (1.14-1.68)	1.78 (1.46-2.16)	<0.01	

CHF, chronic heart failure; CI, confidence interval; CKD, chronic kidney disease; HR, hazard ratio; OR, odds ratio.

Models were adjusted for age, sex, BMI, SBP, DBP, heart rate, EF phenotype, NT-proBNP, CRP, myocardial infarction, hypertension, atrial fibrillation, valvular heart disease, thrombotic complication, diabetes, stroke, anemia, chronic pulmonary disease, liver disease, connective tissue disease, cancer, and medication use of RAASi, spironolactone, diuretics, beta-blockers, digitalis, and nitrates.

**Figure 2:**
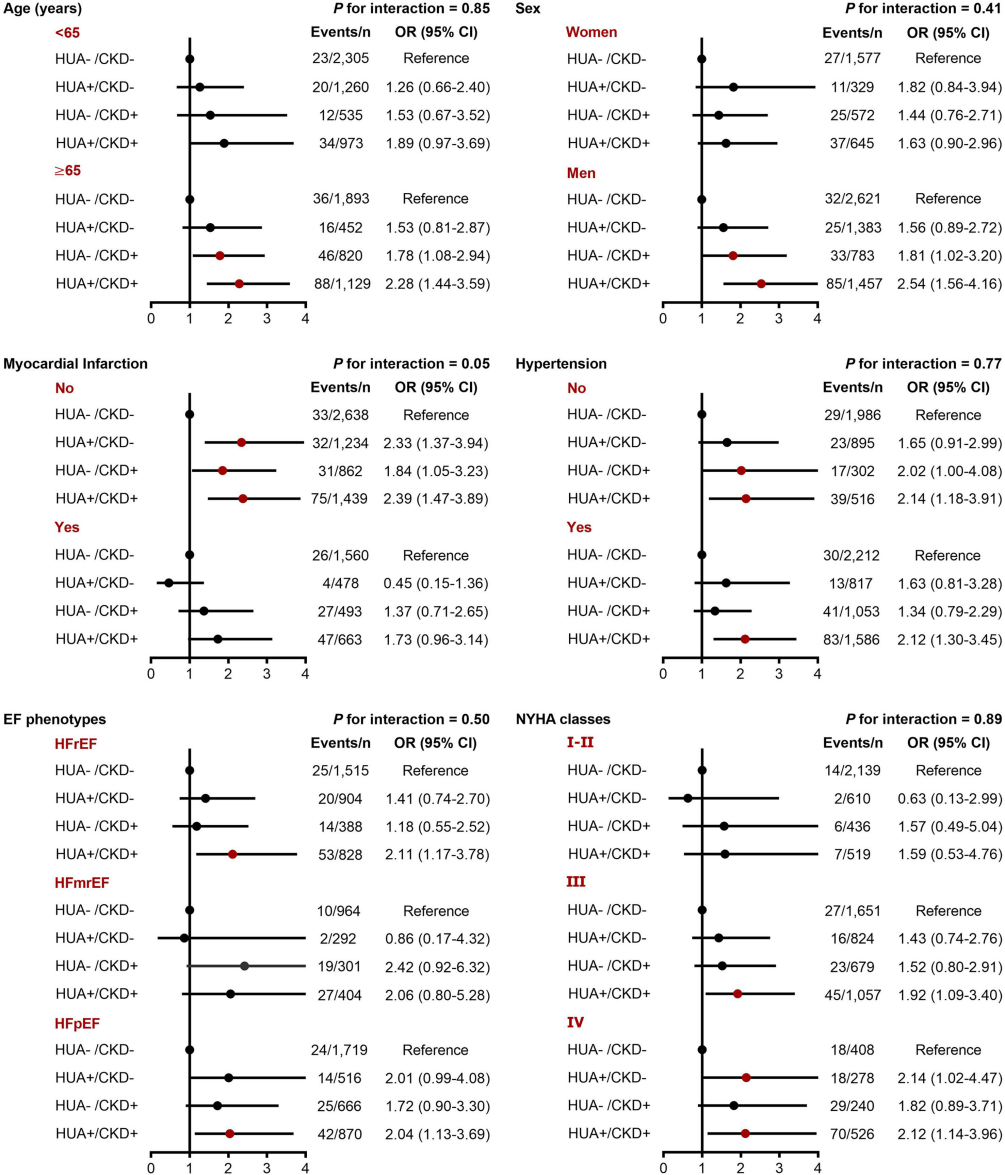
Subgroup analyses for the adjusted OR (95% CI) of hyperuricemia/CKD groups for in-hospital mortality by age, sex, myocardial infarction, hypertension, EF phenotype, and NYHA classification. CI, confidence interval; CKD, chronic kidney disease; EF, ejection fraction; HFmrEF, heart failure with mildly reduced ejection fraction; HFpEF, heart failure with preserved ejection fraction; HFrEF, heart failure with reduced ejection fraction; HUA, hyperuricemia; NYHA, New York Heart Association; OR, odds ratio.

**Figure 3:**
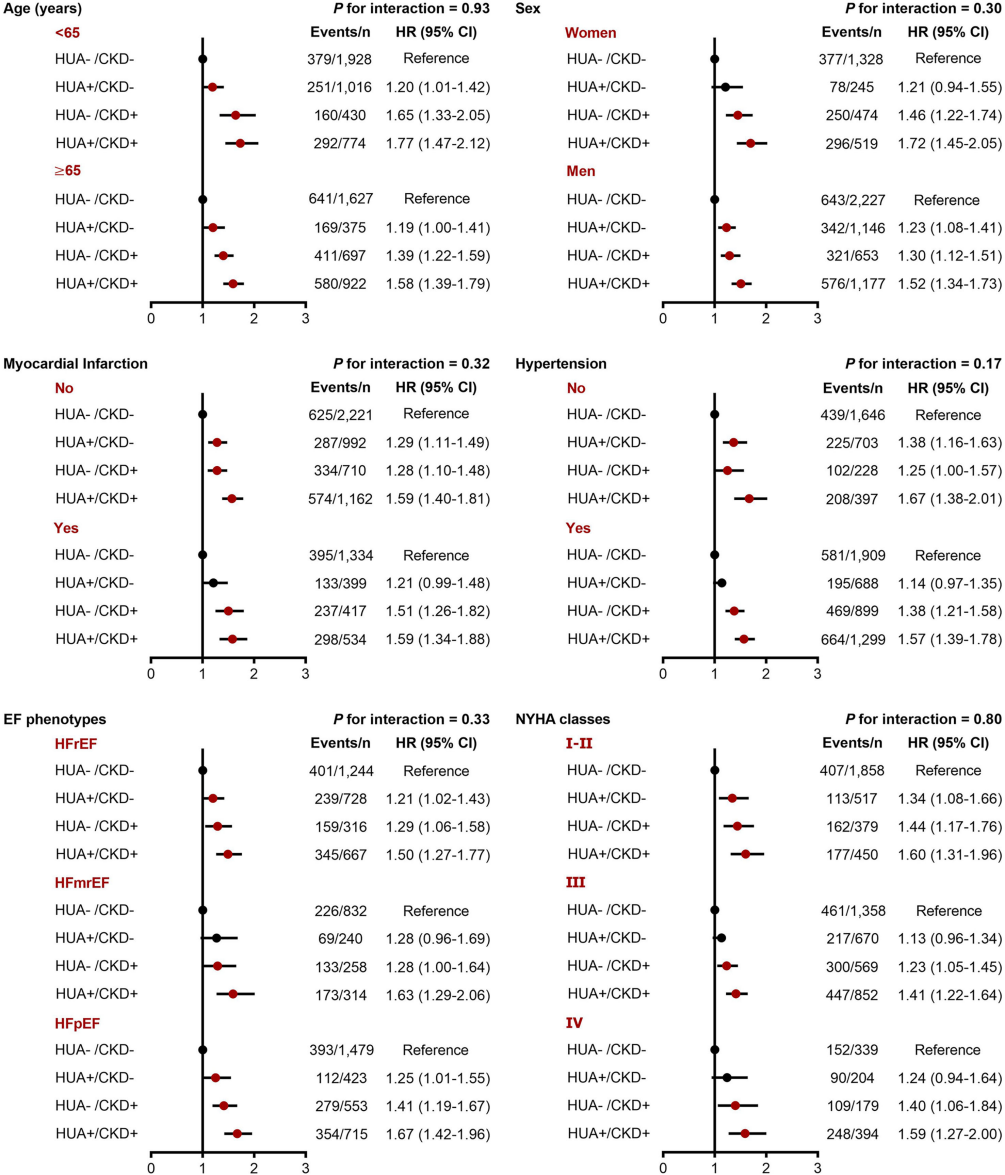
Subgroup analyses for the adjusted HR (95% CI) of hyperuricemia/CKD groups for long-term mortality by age, sex, myocardial infarction, hypertension, EF phenotype, and NYHA classification. CI, confidence interval; CKD, chronic kidney disease; EF, ejection fraction; HFmrEF, heart failure with mildly reduced ejection fraction; HFpEF, heart failure with preserved ejection fraction; HFrEF, heart failure with reduced ejection fraction; HR, hazard ratio; HUA, hyperuricemia; NYHA, New York Heart Association.

In the subgroup analyses, we did not find significant interactions between hyperuricemia/CKD groups and age, sex, hypertension, EF phenotype, and NYHA classification in relation to both in-hospital mortality and long-term mortality (*P*-interaction >0.10 for all). However, the association between hyperuricemia/CKD groups and in-hospital mortality appeared to be more pronounced in CHF patients without myocardial infarction (*P*-interaction =0.05) (**Figures 2**, **3**).

## Discussion

In this retrospective cohort study, we found that hyperuricemia and CKD were individually and cumulatively associated with increased risk of in-hospital mortality and long-term mortality in patients with CHF. These findings persisted after adjusting for a wide spectrum of confounding factors and in sensitivity analyses. Moreover, the risks of both in-hospital mortality and long-term mortality were proportional to the serum UA level and risk classification of CKD progression. However, serum UA had a weaker association with in-hospital mortality in patients with eGFR <60 ml/min/1.73m^2^, and a weaker association with long-term mortality in patients with proteinuria.

In recent years, the prognostic role of hyperuricemia in patients with heart failure has garnered particular interest. Findings from Metabolic Exercise Cardiac Kidney Index score database showed that each 60 μmol/L increase in UA was associated with 12% increased risk of mortality in patients with HFrEF (36), whereas Mantovani et al. found that hyperuricemia increased the mortality risk by 37% in general patients with CHF (12). CKD has a bidirectional relationship with hyperuricemia (37, 38), and may mediate the association between hyperuricemia and health outcomes (16). However, to the best of our knowledge, only a few studies examined the combined effect of hyperuricemia and CKD on mortality risk in patients with CHF, but generated inconsistent results with our study (19–21). For example, a study of 1,260 systolic heart failure patients from the Beta-Blocker Evaluation of Survival Trial found that hyperuricemia was associated with a 1.4-fold higher risk of mortality in patients without CKD, but not in those with CKD (19). However, this previous study only included patients with NYHA class III and IV systolic heart failure who were screened for participation in a clinical trial, while our study was performed in a real-world setting and included patients with all phenotypes of CHF. Thus, the study patients in our study might be more representative. In another cohort study including 4,652 patients with CHF, the association between serum UA and all-cause mortality was significantly modified by eGFR, and hyperuricemia was not associated with outcome in patients with CKD (20). Vaduganathan et al. also found that serum UA (per 300 μmol/L increase) was not associated with increased hazard of all-cause mortality in CHF patients with eGFR <30 ml/min/1.73m^2^ (21). Notably, the definition of CKD in these studies did not account for proteinuria, which could not provide complete information on renal dysfunction. In the current study, we used a more complete definition of CKD, and observed that elevated serum UA was associated with increased risk of mortality in CHF patients with and without CKD, low eGFR, and proteinuria. Taken together, our study provided a more comprehensive understanding regarding the association between hyperuricemia, CKD, and mortality in patients with CHF by using well-characterized information and larger sample size.

It is well-known that CKD contributes to worse outcomes in patients with CHF by uremic toxins, fluid overload, enhancing systemic inflammation and oxidative stress, and activating the sympathetic nervous system and renin-angiotensin-aldosterone system (39–41). Several mechanisms may explain why the presence of both CKD and hyperuricemia cause a cumulative increase in risk of mortality. First, hyperuricemia may cause renal tubular injury, and accelerate the progression of CKD (42, 43), hence increases mortality risk. Second, increased xanthine oxidase activity, a major cause of hyperuricemia, can promote oxidative stress and upregulation of inflammatory cytokines, which further result in cardiac fibrosis and left ventricular dysfunction (44). It is still conflicting that whether UA is a direct risk factor of cardiovascular outcomes or just a passive marker of xanthine oxidase activity. However, in the current study, the risk of long-term mortality related to hyperuricemia was similar between patients with eGFR ≥60 ml/min/1.73m^2^ (hyperuricemia mainly caused by reduced UA excretion) and patients with eGFR <60 ml/min/1.73m^2^ (hyperuricemia mainly caused by increased xanthine oxidase activity). Recent studies of sodium-glucose cotransporter 2 inhibitors (SGLT2i) have suggested that the beneficial effect of SGLT2i on heart failure outcomes is mediated by their enhancement of renal UA excretion (45). Thus, elevated serum UA may directly contribute to the adverse outcomes in patients with CHF. Potential mechanisms include UA-induced vascular fibrosis, vascular inflammation, endothelial dysfunction, and nitric oxide reduction (46).

The findings of our study provide important clinical implications. Hyperuricemia is commonly recognized as a bystander in patients with CHF and CKD. However, our study showed that hyperuricemia and CKD cumulatively increased the risk of mortality in patients with CHF. This suggests that measurement of serum UA concentration might provide useful information on the assessment of outcome risk, regardless of kidney function. Moreover, these findings support the importance of including UA management in the treatment strategies of CHF regardless of the cause of hyperuricemia (increased production or decreased excretion). There is also a need to strengthen the patient education regarding limiting their intake of purine-rich food. Several novel medications for heart failure (e.g., angiotensin receptor neprilysin inhibitors, SGLT2i) have been demonstrated to lower serum UA concentration, which may be associated with improved outcomes (45, 47). Further clinical trials are warranted to assess the effect of UA-lowering treatment on adverse health outcomes in patients with CHF, CKD, and hyperuricemia.

The strengths of our study included the cohort design, large sample size, and well-characterized data on a broad spectrum of biological and demographic fields. However, several limitations should be noted. First, our study was conducted in a single center, although, over 70% of the study patients were from other 30 provinces of China. Therefore, the patients included in our study were still nationally representative, but may not be completely generalizable to other countries. Second, 14.12% of the patients were lost to follow-up, which may have introduced bias into the long-term mortality analysis. However, the proportion of CKD and many other basic characteristics were comparable between patients who lost to follow-up and those included in the long-term mortality analysis (**Supplementary Table 1**). Therefore, the bias could be small. Third, due to the limitation of telephonic interview, we did not collect information on specific causes of death in all study patients. Fourth, data on serum UA, eGFR, and proteinuria were only obtained at baseline and were not assessed over time. Since these are all modifiable factors, the longitudinal trajectories of these parameters and their association with mortality in patients with CHF warrant investigation in the future.

In conclusion, hyperuricemia and CKD individually and cumulatively increase the risk of in-hospital mortality and long-term mortality in patients with CHF. These findings provided further evidence demonstrating the combined effect of hyperuricemia and CKD on heart failure outcomes, and highlighted the importance of the management of serum UA in CHF patients, with or without CKD.

## Data availability statement

The raw data supporting the conclusions of this article will be made available by the authors, without undue reservation.

## Ethics statement

The studies involving human participants were reviewed and approved by the ethics committee of the Chinese People’s Liberation Army General Hospital. The ethics committee waived the requirement of written informed consent for participation.

## Author contributions

Conceptualization: CW, WD, YB, and KH; Data curation: CW, HC, YZ, RW, DZ, LC, CR, QZ, ZL, YD, and JX; Formal analysis: CW and HC; Funding acquisition: KH; Investigation: CW, HC, and YZ; Methodology: CW and HC; Project administration: KH; Writing-original draft: CW and HC; Writing-review and editing: All authors. All authors contributed to the article and approved the submitted version.
