# Supplementary material for: Joint association of hyperuricemia and chronic kidney disease with mortality in patients with chronic heart failure
Source: Front Endocrinol (Lausanne). 2023 Apr 5;14:1131566. doi: 10.3389/fendo.2023.1131566 (PMC10113528; doi:10.3389/fendo.2023.1131566)
Supplement: Supplementary file 1 [file Table_1.docx]

**Joint Association of Hyperuricemia and Chronic Kidney Disease with Mortality**

**in Patients with Chronic Heart Failure**

Chi Wang, MD, Hebin Che, MSE, You Zhou, MD, Ruiqing Wang, PhD, Di Zhu, MD, Liting Cheng, MD, Chongyou Rao, MD, Qin Zhong, MD, Zongren Li, PhD, Yongjie Duan, MD, Jiayu Xu, MD, Wei Dong, MD, PhD, Yongyi Bai, MD, PhD, Kunlun He, MD, PhD

**Supplementary Material**

**Table of Contents**

[**Supplementary Table 1.** Baseline comparison between patients included in the long-term mortality analysis and patients lost to follow-up 2](#_Toc129394306)

[**Supplementary Table 2.** Baseline characteristics of patients included in the long-term mortality analysis according to hyperuricemia/CKD groups 4](#_Toc129394307)

[**Supplementary Table 3.** Risk of in-hospital CV mortality and long-term CV mortality according to hyperuricemia/CKD groups 6](#_Toc129394308)

# Supplementary Table 1. Baseline comparison between patients included in the long-term mortality analysis and patients lost to follow-up

| **Variables** | **Patients lost to**  **follow-up** | **Patients included**  **in the long-term mortality analysis** | ***P* value** |
| --- | --- | --- | --- |
| No. of patients | 1,323 | 7,769 |  |
| Age, mean ±SD, y | 59.54±14.10 | 62.90±14.56 | <0.01 |
| Men, No. (%) | 866 (65.46) | 5,203 (66.97) | 0.28 |
| Hyperuricemia, No. (%) | 569 (43.01) | 3087 (39.73) | 0.02 |
| CKD, No. (%) | 454 (34.32) | 2823 (36.34) | 0.16 |
| Hyperuricemia/CKD group |  |  | 0.01 |
| Hyperuricemia-/CKD- | 584 (44.14) | 3,555 (45.76) |  |
| Hyperuricemia+/CKD- | 285 (21.54) | 1,391 (17.90) |  |
| Hyperuricemia-/CKD+ | 170 (12.85) | 1,127 (14.51) |  |
| Hyperuricemia+/CKD+ | 284 (21.47) | 1,696 (21.83) |  |
| BMI, mean ±SD, kg/m^2^ | 24.82±4.45 | 25.11±4.14 | 0.02 |
| SBP, mean ±SD, mmHg | 128.71±23.57 | 130.90±22.38 | <0.01 |
| DBP, mean ±SD, mmHg | 76.25±14.29 | 75.69±14.02 | 0.19 |
| Heart Rate, mean ±SD, bpm | 82.73±17.62 | 82.79±18.12 | 0.92 |
| EF phenotypes, No. (%) |  |  | <0.01 |
| HFrEF | 568 (42.93) | 2,955 (38.04) |  |
| HFmrEF | 259 (19.58) | 1,644 (21.16) |  |
| HFpEF | 496 (37.49) | 3,170 (40.80) |  |
| NYHA class, No. (%) |  |  | <0.01 |
| Ⅱ | 471 (35.60) | 3,204 (41.24) |  |
| Ⅲ | 651 (49.21) | 3,449 (44.39) |  |
| Ⅳ | 201 (15.19) | 1,116 (14.36) |  |
| **Laboratory test** |  |  |  |
| UA, median (IQR), μmol/L | 393.40 (315.85, 490.15) | 384.40 (308.30, 476.40) | <0.01 |
| eGFR, median (IQR), ml/min/1.73m^2^ | 78.10 (55.75, 93.52) | 75.42 (53.29, 91.59) | <0.01 |
| NT-proBNP, median (IQR), pg/mL | 2602.0 (961.2, 6818.0) | 1990.0 (797.5, 5382.0) | <0.01 |
| CRP, No. (%) |  |  | <0.01 |
| <1 mg/dL | 762 (71.28) | 5,129 (74.86) |  |
| 1-3 mg/dL | 190 (17.77) | 929 (13.56) |  |
| ≥3 mg/dL | 117 (10.94) | 793 (11.57) |  |
| Proteinuria, No. (%) | 206 (16.57) | 1,089 (15.04) | 0.16 |
| **Comorbidities** |  |  |  |
| Myocardial infarction, No. (%) | 406 (30.69) | 2,684 (34.55) | <0.01 |
| Hypertension, No. (%) | 706 (53.36) | 4,795 (61.72) | <0.01 |
| Atrial fibrillation, No. (%) | 355 (26.83) | 2,405 (30.96) | <0.01 |
| Valvular heart disease, No. (%) | 273 (20.63) | 1,772 (22.81) | 0.08 |
| Thrombotic complications, No. (%) | 64 (4.84) | 433 (5.57) | 0.28 |
| Gout, No. (%) | 21 (1.59) | 174 (2.24) | 0.13 |
| Diabetes, No. (%) | 486 (36.73) | 3,137 (40.38) | 0.01 |
| Stroke, No. (%) | 219 (16.55) | 1,559 (20.07) | <0.01 |
| Anemia, No. (%) | 501 (37.87) | 2,889 (37.19) | 0.64 |
| Chronic pulmonary diseases, No. (%) | 137 (10.36) | 1,028 (13.23) | <0.01 |
| Liver diseases, No. (%) | 171 (12.93) | 1,085 (13.97) | 0.31 |
| Connective tissue diseases, No. (%) | 18 (1.36) | 139 (1.79) | 0.27 |
| Cancer, No. (%) | 32 (2.42) | 267 (3.44) | 0.06 |
| **Medication during hospitalization** |  |  |  |
| UA-lowering agents, No. (%) | 45 (3.40) | 351 (4.52) | 0.07 |
| RAASi, No. (%) | 760 (57.45) | 4,469 (57.52) | 0.96 |
| Spironolactone, No. (%) | 981 (74.15) | 5,496 (70.74) | 0.01 |
| Diuretics, No. (%) | 1,076 (81.33) | 6,211 (79.95) | 0.24 |
| Beta-blockers, No. (%) | 1,027 (77.63) | 6,207 (79.89) | 0.06 |
| Digitalis, No. (%) | 664 (50.19) | 3,536 (45.51) | <0.01 |
| Nitrates, No. (%) | 834 (63.04) | 4,900 (63.07) | 0.98 |
| Abbreviations: BMI, body mass index; bpm, beats per minute; CKD: chronic kidney disease; CRP, C-reactive protein; DBP, diastolic blood pressure; eGFR, estimated glomerular filtration rate; IQR, interquartile range; NT-proBNP: N-terminal pro-B-type natriuretic peptide; RAASi: renin–angiotensin–aldosterone system inhibitors; SBP, systolic blood pressure; SD, standard deviation. | | | |

# Supplementary Table 2. Baseline characteristics of patients included in the long-term mortality analysis according to hyperuricemia/CKD groups

| **Variables** | **Hyperuricemia-**  **/CKD-** | **Hyperuricemia+**  **/CKD-** | **Hyperuricemia-**  **/CKD+** | **Hyperuricemia+**  **/CKD+** | ***P* value** |
| --- | --- | --- | --- | --- | --- |
| No. of patients | 3,555 | 1,391 | 1,127 | 1,696 |  |
| Age, mean ±SD, y | 63.06±13.13 | 56.17±13.82 | 68.26±14.68 | 64.53±15.87 | <0.01 |
| Men, No. (%) | 2,227 (62.64) | 1,146 (82.39) | 653 (57.94) | 1,177 (69.40) | <0.01 |
| BMI, mean ±SD, kg/m^2^ | 24.85±3.81 | 25.94±4.51 | 24.65±4.05 | 25.28±4.44 | <0.01 |
| SBP, mean ±SD, mmHg | 128.43±19.94 | 124.11±19.93 | 140.72±24.45 | 135.11±24.61 | <0.01 |
| DBP, mean ±SD, mmHg | 74.46±12.43 | 76.15±14.14 | 77.20±14.63 | 76.90±16.26 | <0.01 |
| Heart Rate, mean ±SD, bpm | 81.54±17.69 | 84.66±18.00 | 83.21±17.93 | 83.60±19.03 | <0.01 |
| EF phenotypes, No. (%) |  |  |  |  | <0.01 |
| HFrEF | 1,244 (34.99) | 728 (52.34) | 316 (28.04) | 667 (39.33) |  |
| HFmrEF | 832 (23.40) | 240 (17.25) | 258 (22.89) | 314 (18.51) |  |
| HFpEF | 1,479 (41.60) | 423 (30.41) | 553 (49.07) | 715 (42.16) |  |
| NYHA class, No. (%) |  |  |  |  | <0.01 |
| Ⅰ-Ⅱ | 1,858 (52.26) | 517 (37.17) | 379 (33.63) | 450 (26.53) |  |
| Ⅲ | 1,358 (38.20) | 670 (48.17) | 569 (50.49) | 852 (50.24) |  |
| Ⅳ | 339 (9.54) | 204 (14.67) | 179 (15.88) | 394 (23.23) |  |
| **Laboratory test** |  |  |  |  |  |
| UA, mean ±SD, μmol/L | 313.60±65.21 | 509.06±91.42 | 328.70±67.06 | 549.32±125.12 | <0.01 |
| eGFR, median (IQR), ml/min/1.73m^2^ | 87.58 (76.48, 96.82) | 82.81 (71.29, 95.43) | 49.04 (24.19, 58.82) | 39.91 (21.13, 53.81) | <0.01 |
| NT-proBNP, median (IQR), pg/mL | 1167.0 (534.2, 2636.0) | 1691.5 (767.0, 4047.5) | 4209.5 (1585.5, 13092.5) | 5675.0 (2166.0, 15797.0) | <0.01 |
| CRP, No. (%) |  |  |  |  | <0.01 |
| <10 mg/L | 2,385 (78.79) | 976 (80.00) | 715 (68.68) | 1,053 (67.37) |  |
| 10-30 mg/L | 309 (10.21) | 150 (12.30) | 169 (16.23) | 301 (19.26) |  |
| ≥30 mg/L | 333 (11.00) | 94 (7.70) | 157 (15.08) | 209 (13.37) |  |
| Proteinuria, No. (%) | 0 (0) | 0 (0) | 471 (45.46) | 618 (38.77) | <0.01 |
| **Comorbidities** |  |  |  |  |  |
| Myocardial infarction, No. (%) | 1,334 (37.52) | 399 (28.68) | 417 (37.00) | 534 (31.49) | <0.01 |
| Hypertension, No. (%) | 1,909 (53.70) | 688 (49.46) | 899 (79.77) | 1,299 (76.59) | <0.01 |
| Atrial fibrillation, No. (%) | 1,102 (31.00) | 457 (32.85) | 310 (27.51) | 536 (31.60) | 0.03 |
| Valvular heart disease, No. (%) | 923 (25.96) | 362 (26.02) | 166 (14.73) | 321 (18.93) | <0.01 |
| Thrombotic complications, No. (%) | 176 (4.95) | 73 (5.25) | 82 (7.28) | 102 (6.01) | 0.02 |
| Gout, No. (%) | 0 (0) | 58 (4.17) | 0 (0) | 116 (6.84) | <0.01 |
| Diabetes, No. (%) | 1,264 (35.56) | 444 (31.92) | 609 (54.04) | 820 (48.35) | <0.01 |
| Stroke, No. (%) | 672 (18.90) | 179 (12.87) | 324 (28.75) | 384 (22.64) | <0.01 |
| Anemia, No. (%) | 960 (27.00) | 284 (20.42) | 690 (61.22) | 955 (56.31) | <0.01 |
| Chronic pulmonary diseases, No. (%) | 433 (12.18) | 147 (10.57) | 199 (17.66) | 249 (14.68) | <0.01 |
| Liver diseases, No. (%) | 448 (12.60) | 202 (14.52) | 160 (14.20) | 275 (16.21) | <0.01 |
| Connective tissue diseases, No. (%) | 47 (1.32) | 22 (1.58) | 28 (2.48) | 42 (2.48) | <0.01 |
| Cancer, No. (%) | 143 (4.02) | 39 (2.80) | 38 (3.37) | 47 (2.77) | 0.05 |
| **Medication after discharge** |  |  |  |  |  |
| UA-lowering agents, No. (%) | 0 (0) | 91 (6.54) | 0 (0) | 260 (15.33) | <0.01 |
| RAASi, No. (%) | 1,539 (43.29) | 679 (48.81) | 472 (41.88) | 667 (39.33) | <0.01 |
| Spironolactone, No. (%) | 2,217 (62.36) | 1,091 (78.43) | 571 (50.67) | 962 (56.72) | <0.01 |
| Diuretics, No. (%) | 1,401 (39.41) | 795 (57.15) | 548 (48.62) | 1,054 (62.15) | <0.01 |
| Beta-blockers, No. (%) | 2,354 (66.22) | 1,047 (75.27) | 770 (68.32) | 1,162 (68.51) | <0.01 |
| Digitalis, No. (%) | 1,259 (35.41) | 683 (49.10) | 249 (22.09) | 549 (32.37) | <0.01 |
| Nitrates, No. (%) | 1,495 (42.05) | 505 (36.30) | 553 (49.07) | 776 (45.75) | <0.01 |
| Abbreviations: BMI, body mass index; bpm, beats per minute; CKD: chronic kidney disease; CRP, C-reactive protein; DBP, diastolic blood pressure; eGFR, estimated glomerular filtration rate; IQR, interquartile range; NT-proBNP: N-terminal pro-B-type natriuretic peptide; RAASi: renin–angiotensin–aldosterone system inhibitors; SBP, systolic blood pressure; SD, standard deviation; UA, uric acid. | | | | | |

# Supplementary Table 3. Risk of in-hospital CV mortality and long-term CV mortality according to hyperuricemia/CKD groups

|  | **Hyperuricemia/CKD Groups** | | | |
| --- | --- | --- | --- | --- |
|  | **Hyperuricemia-**  **/CKD-** | **Hyperuricemia+**  **/CKD-** | **Hyperuricemia-**  **/CKD+** | **Hyperuricemia+**  **/CKD+** |
| **In-hospital CV mortality, OR (95% CI)** | | | | |
| Cases, No. | 45 | 32 | 25 | 80 |
| Incidence rate, % | 1.07 | 1.87 | 1.85 | 3.81 |
| Model 1 | 1 (Reference) | 1.76 (1.11-2.78) | 1.74 (1.06-2.84) | 3.65 (2.53-5.28) |
| Model 2 | 1 (Reference) | 2.03 (1.27-3.23) | 1.56 (0.95-2.56) | 3.50 (2.42-5.08) |
| Model 3 | 1 (Reference) | 1.58 (0.98-2.56) | 0.97 (0.56-1.70) | 1.74 (1.12-2.71) |
| **Long-term CV mortality, HR (95% CI)** | | | | |
| Cases, No. | 493 | 244 | 259 | 437 |
| Incidence rate, per 1000-person-year | 26.76 | 34.78 | 56.82 | 67.97 |
| Model 1 | 1 (Reference) | 1.30 (1.11-1.51) | 2.08 (1.79-2.42) | 2.47 (2.17-2.81) |
| Model 2 | 1 (Reference) | 1.53 (1.31-1.79) | 1.81 (1.55-2.11) | 2.37 (2.08-2.70) |
| Model 3 | 1 (Reference) | 1.34 (1.15-1.58) | 1.44 (1.22-1.69) | 1.66 (1.43-1.92) |
| Abbreviations: CKD: chronic kidney disease; CI: confidence interval; CV: cardiovascular; HR, hazard ratio; OR, odds ratio.  Model 1 was crude model. Model 2 was adjusted for age and sex. Model 3 was further adjusted for BMI, SBP, DBP, heart rate, EF phenotype, NT-proBNP, CRP, myocardial infarction, hypertension, atrial fibrillation, valvular heart disease, thrombotic complication, diabetes, stroke, anemia, chronic pulmonary disease, liver disease, connective tissue disease, cancer, and medication use of RAASi, spironolactone, diuretics, beta-blockers, digitalis, and nitrates. | | | | |
